# Hybridization with *Fagopyrum cymosum* Meisn. as a way to make cultivated Tartary buckwheat (*F. tataricum* Gaertn.) with grain characteristics typical for common buckwheat (*F. esculentum* Moench.)

**DOI:** 10.1270/jsbbs.21086

**Published:** 2022-06-17

**Authors:** Ivan N. Fesenko, Nikolay I. Bondarev, Olga V. Rezunova, Darya E. Evsyuticheva, Aleksey N. Fesenko

**Affiliations:** 1 Lab of Buckwheat Breeding, Federal Scientific Center of Grain Legumes and Groats Crops, 302502, p/o Streletskoe, Orel, Russia; 2 Department of Industrial Chemistry and Biotechnology, Orel State University named after I.S. Turgenev, 302026, Komsomolskaya 95, Orel, Russia

**Keywords:** buckwheat, *Fagopyrum tataricum*, interspecific hybridization, seed type

## Abstract

Compared to common buckwheat (*F. esculentum*), Tartary buckwheat (*F. tataricum*) is very polymorphic in the type of seeds, but a seed type which is typical for *F. esculentum*, i.e. triangular seeds with flat sides and clear ribs, has been not found among the polymorphism. However, such seed type is typical for wild species *F. cymosum* which produces fertile hybrids in crosses with *F. tataricum*. Embryo rescue based interspecific cross *F. esculentum* × *F. cymosum* allowed reveal functional allelism of the genes determining the similar morphs of these species’ seeds, i.e. the seed type resulted from mutation(s) at same gene. The gene can be assigned as *TAN* (triangular). Variation for the seed shell thickness among recessives for the *TAN* carrying about 12% of *F. tataricum* genome, together with the shell thickness of a seed from the F_1_ hybrid *F. esculentum* × *F. cymosum* compared to ones of the parents, suggests there are some genes influencing seed shell thickness. Also, it was supported by analyses of seeds characteristics of Tartary-based forms with some share of *F. cymosum* genetic material. In addition, cross *F. tataricum* × *F. cymosum* looks like an effective tool to increase 1000-seed weight of Tartary buckwheat-based breeding material.

## Introduction

Two buckwheat species are cultivated as grain or groats crops, *Fagopyrum esculentum* Moench. (common buckwheat) and *F. tataricum* Gaertn. (Tartary buckwheat). Outside Southeast Asia the buckwheat cultivation is represented by only one biological species, *F. esculentum*. In Russia, introducing *F. tataricum* into agricultural practice is currently considered (Fesenko *et al.* 2017). This species has several advantages compared to *F. esculentum*, such as a much higher content of flavonoids in the grain (Mukasa *et al.* 2009, Vogrinčič *et al.* 2013, Zhu 2016). However, Tartary buckwheat grain usually requires other processing technologies compared to common buckwheat grain, i.e. grinding of whole grain instead of shelling (Klimova *et al.* 2020, Li *et al.* 2019). However, on the other hand, the species has wide polymorphism for the seed (grain) types, and the different types are suitable for different technologies. The variability of *F. tataricum* for the seed shape is classified into four types (Hirose *et al.* 1995, Wang and Campbell 2007): 1) type ‘notched’, the seeds with sculptured formations on surface, which are typical for wild or weedy forms of the species; 2) type ‘round’, seeds without sculptured formations on pericarp which length and width are approximately equal, the result of a recessive mutation *smk* (Fesenko 2012); 3) type ‘slender’, a form which is mainly same with ‘round’, but the seeds are prolonged, i.e. the length is twice of width; and 4) ‘rice’ type, seeds without sculptured formations on pericarp, usually with split instead of groove on the seed sides, result of combination of two recessive genes, *smk* and *spl* (Fesenko 2012). The easy-shelling ‘rice’ Tartary buckwheat is considered the best for breeding varieties suitable for groats production (Li *et al.* 2019, Mukasa *et al.* 2007, Wang and Campbell 2007).

However, some other approaches to obtain Tartary buckwheat with easy-shelling grain also could be considered. One of them is interspecific hybridization which is promising way to obtain Tartary buckwheat with seeds similar to ones of common buckwheat. It means not only the seed shape but also a thousand seed weight (TSW) since its upper limit within *F. tataricum* variation is about 23 g (Li *et al.* 2019). Perhaps, it will make it possible to breed Tartary buckwheat which is best suited for groats production.

Gene exchange between *F. tataricum* and *F. esculentum* is absolutely impossible due to strong post-zygotic barriers (Samimy *et al.* 1996). However, experiments on interspecific hybridization in genus *Fagopyrum* reveal the possibility to use *F. cymosum*, a species possessing type of seeds similar to *F. esculentum*, as a source of additional genetic polymorphism for Tartary buckwheat. Exchange of genetic material between *F. tataricum* and *F. cymosum* is possible at both ploidy levels; at tetraploid level an artificial species *F. giganteum* which is allopolyploid *F. tataricum* (4x) × *F. cymosum* (4x) was used as a “bridge” for the interspecific genes exchange (Fesenko *et al.* 2001, Fesenko and Fesenko 2010, Krotov and Dranenko 1973).

Trying to conduct the allelism test for the genes that control the similar shape of the seeds of the two species we obtained interspecific hybrid *F. esculentum* × *F. cymosum*. Also, we have got hybrids BC_1_F_2_ *F. cymosum* × *F. giganteum* and have selected several plants with *F. cymosum*-like triangular seeds. All the hybrids were characterized in comparison with parents and some other forms of the buckwheat species. The objective of the paper was to describe and discuss the results.

## Materials and Methods

### Plant material (accessions and hybrids)

#### *Fagopyrum* species 

*F. cymosum*: a tetraploid (4x = 32) accession k-4231 from collection of N.I. Vavilov’s Plant Genetic Resources Center (VIR), St. Petersburg.

*F. tataricum*: diploid (2x = 16) accessions of VIR collection k-2, k-3, k-4, k-8, k-10, k-11, k-12, k-14, k-21, k-22, k-25, k-26, k-29, k-107, k-73 (type ‘notched’), k-17, k-77, k-101 (type ‘round’) and k-103 (type ‘rice’); several accessions of hybrid origin, i.e. 1 accession F_8_ (k-17 × k-66) (type ‘round’) and 9 accessions F_6_ k-103 × k-17 (type ‘rice’); tetraploid accession k-108 (type ‘round’).

*F. esculentum*: diploid (2x = 16) varieties Dikul, Devyatka, Dialog, Temp and Dasha bred in FSC of Legumes and Groats Crops; tetraploid (4x = 32) accession with winged seeds which was used for interspecific hybridization with k-4231 *F. cymosum*.

#### Interspecific hybrids 

*F. giganteum*, an artificial species with allopolyploid genome, was first obtained by Krotov and Dranenko (1973) by cross between tetraploid accessions of *F. tataricum* and *F. cymosum*. All plants of the species were pin-flowered. We used in the work the hybrids *F. tataricum* (k-108) × thrum *F. cymosum* (k-4231) which were obtained later (Fesenko 2005) and represented by both pin and thrum-flowered plants. These hybrids are similar to the *F. giganteum* in terms of genome composition since they are resulted from same interspecific cross, and could be considered as a part of this species. However, only the thrum-flowered plants is suitable as a pollen parent for hybridization with *F. cymosum*.

BC_1_F_2_ (*F. cymosum*, k-4231 × *F. giganteum*) were obtained using conventional crossing *F. cymosum*, pin × thrum *F. giganteum* with following backcrossing F_1_, thrum × pin *F. cymosum*. The resulting population was heterostylous. Second generation of the backcrosses was used in the work. BC_1_F_2_ hybrids were segregated in seed type into two types: triangular *F. cymosum*-like seeds and notched *F. giganteum*-like seeds. Only plants with triangular seeds without sculptured formations on its surfaces, i.e. typical for *F. cymosum*, were taken for this work. All the BC_1_F_2_ hybrids were late-maturing (period from sowing to ripening was 111–115 days). Parental forms of *F. cymosum* and *F. giganteum* have similar characteristics, 121 and 109 respectively. The seeds used in this work ripened in September.

A new artificial species *F. hybridum* (productive and relatively early ripening progenies of hybrids *F. tataricum* × *F. giganteum*) (Fesenko and Fesenko 2010). Different lineages of *F. hybridum* produce seeds of both ‘round’ and ‘notched’ types. For this work it was taken the accession with ‘round’ seeds only.

F_1_ hybrid clone (*F. esculentum* × *F. cymosum*, k-4231) was obtained using embryo rescue technique (see Experimental approaches). The plants sometimes formed seeds (without pollination by other plants) which usually were die off, but two of the seeds were filled. The seeds were germinated, and its hulls were dried and used for description in the work.

### Experimental approaches

#### Evaluating the seeds characteristics 

To measure the thickness of the shells, seed sections were cut with a blade. The sections were photographed with an AxioCam MRc5 camera (AxioImiger microscope. Al, Carl Zeiss). The measurements were made using the Axio Vision software. The thickness of the shell at the cross section was measured in three points: near the rib, in the middle of a side, and between these points. The average value was calculated and included in statistics. At least two seeds of every sample were studied. If a “cluster” was represented by single accession only, 10 seeds were analyzed.

To evaluate the thousand seeds weight 100 typical well developed seeds were weighed.

To evaluate the seed shell rate 100 well developed seeds were weighed and germinated in a Petri dish. Then, the empty shells were collected, dried and weighed.

To test the significance of the detected differences it was used t-statistics.

#### Embryo rescue technique 

The cross *F. esculentum* × *F. cymosum* at both diploid and tetraploid levels is impossible using conventional hybridization due to strong barriers at the stage of hybrid seeds development: after pollination using pollen of another species the seeds start to develop but die off after about 7–9 days (Suvorova *et al.* 1994). We crossed tetraploid accessions of *F. esculentum* and *F. cymosum* in September 2018. The forming hybrid seeds 6–7 days after pollination were used in the experiment. Hybrid embryos were removed from pericarps sterilized using 3% hydrogen peroxide solution or 0.05% chlorhexidine bigluconate solution during 5 min, and washed with sterile distilled water. Then these hybrid embryos were transferred to the modified Murashige-Skoog (MS) medium (Murashige and Skoog 1962) containing 3% sucrose, 500 mg/l of casein hydrolyzate, plant growth regulators 6-benzylaminopurine (BA), 2 mg/l, and 3-indoleacetic acid (IAA), 0.2 mg/l, in addition to mineral salts and vitamins. The formed embryoids were transferred to hormone-free MS medium. The grown microshoots were rooted using MS medium supplemented with 0.5 mg/L of 3-indolylbutyric acid (IBA). The cultivation was carried out at a temperature of 25 ± 1°C, illumination of 2500 lx, an air humidity of 70%, and 16-hour light day length. Rooted microshoots were removed from test tubes and washed with water. Then they were planted in containers with soil and grown in a greenhouse.

## Results

### Allelism test for the genes controlling seed shape in *F. esculentum* and *F. cymosum*

**Fig. 1.**
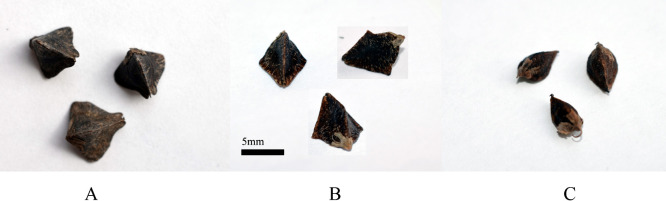
Seeds of A) *F. esculentum* (4x), B) F_1_ *F. esculentum* (4x) × *F. cymosum* (4x) (photos of one seed from different sides), C) *F. cymosum* (4x).

*F. esculentum* and *F. cymosum* produce morphologically similar triangular seeds with flat sides and clear ribs (Fig. 1). The gene controlling this trait in *F. cymosum* behaves as recessive in cross with *F. tataricum*. So, in cross between *F. cymosum* and *F. tataricum* with round seeds (genotype *smk/smk*) the notched type was restored. It was not strongly surprising, since both the seed type are lost sculptured formations on its covers, but they have different morphology in some other aspects.

In our work the functional allelism test of genes determining seed type in *F. cymosum* and *F. esculentum* was conducted using direct cross between representatives of these species. Hybrids *F. esculentum* × *F. cymosum* at both diploid and tetraploid levels were obtained many times (Rumyantseva *et al.* 1995, Suvorova *et al.* 1994, Ujihara *et al.* 1990). However, the hybrids was failed to produce any seeds. Using standard embryo resque technique we obtained one F_1_ hybrid between tetraploid accessions *F. esculentum* and *F. cymosum*. This hybrid set several seeds. Most of them was died off at early developmental stage, but two seeds were successfully filled. The seeds had no any sculptured formations, as in wild forms of *F. tataricum*. It had even sides and clear ribs, with wings typical for the parent form of *F. esculentum* (Fig. 1). So, the morphs of the seeds of *F. esculentum* and *F. cymosum* looks like results of the same mutational event, or independent mutations at same gene. The gene can be assigned as *TAN* (triangular).

### Thousand seeds weight (TSW)

Diploid *F. tataricum* in our work manifests variation for the TSW within 11.7–22.5 g (Table 1). Minimal values, 11.7–17.7 g, are characteristics of samples with the ‘rice’ type of seeds. Tetraploid accession k-108 shows TSW = 25.5 g. Interspecific hybridization allows obtain plants and varieties with TSW about 30 g and more. So, there is a long-term lineage of hybrid origin (backcrosses *F. tataricum* × *F. giganteum*) with TSW = 41.1 g. Therefore, the trait is under additive control (at least, in part), and the interspecific cross looks as most perspective approach to improve this character. Interspecific hybrids with triangular seeds demonstrate TSW up to 29.9 g.

### Seed shell rate

Variation for the seed shell rate is shown in Table 1. Minimal values (13.6–18.4%) are typical for the ‘rice’ type. Maximal values characterize tetraploid accessions and interspecific hybrids. Seed shell rate, as a rule, manifests a negative correlation with TSW. The value of the correlation coefficient (r) depends on type of material. So, for ‘notched’, and for ‘rice’ samples of *F. tataricum* it was r = –0.25 and –0.53, respectively. The correlation coefficient for BC_1_F_3_ *F. cymosum* × *F. giganteum* was –0.70; commertial diploid varieties of *F. esculentum* show r = –0.92.

The trait depends on both shell thickness and size of kernel compared to size of shell. To characterize development of shell itself we used measurement of its thickness.

### Seed shell thickness

#### F_1_ hybrid *F. esculentum* (4x) × *F. cymosum* (4x) 

The hybrid is an example of genotype *Fc-tan Fc-tan/Fe-tan Fe-tan*, and forms seeds of triangular type which are similar with ones of parental species. The common buckwheat tetraploid used in the interspecific crossing forms seeds with a shell 69.0 ± 2.1 μm thick. Diploid common buckwheat varieties show the same grades (Table 2). So, polyploidization does not affect this parameter in *F. esculentum*. The thickness of seed shell of tetraploid *F. cymosum* (k-4231) was 88.8 ± 2.9 μm. The thickness of shell of the seeds developed on the F_1_ hybrid (*F. esculentum* × *F. cymosum*, k-4231) was 97.9–101.3 μm. The values are within 2σ interval of the variation of k-4231 *F. cymosum*. So, it not significantly differs from *F. cymosum* (P < 0.05). It looks like the *F. esculentum* did not add into the hybrid genotype any alleles which could increase the shell thickness.

#### Constant hybrid *F. tataricum* (4x) × *F. cymosum* (4x), i.e. allopolyploid *F. giganteum* 

The cross sharply changes a type of seeds in hybrids compared to both parents. Sculptured formations on surface of seed are restored. Seed shell of *F. giganteum* (141.9 ± 6.1 μm) is much thicker compared to parental forms of *F. tataricum* (111.4 ± 5.5 μm) and *F. cymosum* (88.8 ± 2.9 μm).

#### *F. hybridum* 

*F. hybridum* and the accession k-108 *F. tataricum* (one of the *F. hybridum* parental form) produce seeds of the type ‘round’ (homozygote for the recessive allele *smk*). Comparison between the samples allows evaluate the influence of the interaction of genes of *F. tataricum* and *F. cymosum* affecting the thickness of the shell on the background of the same genotype that determines the type of seeds (homozygote for the recessive *smk*). *F. hybridum* forms seeds with much thicker shell compared to k-108 (t = 3.52; P < 0.01). *F. hybridum* carries up to 25% of *F. cymosum* genetic material. Therefore, introgression of some genes of *F. cymosum* into *F. tataricum* genome results in some hypertrophy of seed shell. Probably, these species carry different genes influencing the seed shell thickness.

#### BC_1_F_2_ hybrids *F. cymosum* × *F. giganteum* 

The hybrids carry approximately 12% of *F. tataricum* genetic material on the genetic background of *F. cymosum*. They are significantly varied in the thickness of seed shell. Maximal value among the hybrids was 151.2 μm. One of the plants produced seed shell of same thickness compared to *F. cymosum* (86.6 ± 3.7 μm and 88.8 ± 2.9 μm, respectively, t = 0.26). It suggests possibility for selection on this trait, due to the seed shell thickness in the hybrids depends on the presence in the hybrid genotype of certain genes of *F. tataricum*. The variation sometimes was very much within a plant: the maximal range was 77.7–147.7 μm. It suggests the transferred genes sometimes work instable. Among nine plants analyzed eight formed seeds with more thick shell compared to *F. cymosum*. However, only three of them showed a t-test confirmed excess.

According to the results obtained, the use of interspecific hybridization can increase the thickness of the shell by about 10%, on average. Probably, it can increase the seed shell rate up to 2–3% (if not taken into account other possible differences between the seeds of different accessions derived from such crosses). Seed shell rates of the hybrids more than 30% exceed one of *F. cymosum* (26.7–32.9% vs. 21.4%). It can be additionally explained by the fact that in hybrids compared to parental form of *F. cymosum* the kernel, as a rule, does not get all the space inside the shell.

#### Other types of *F. tataricum* (for comparison) 

Seeds of ‘rice’ type (recessive homozygous at both *SMK* and *SPL*) are characterized by minimal shell thickness (Table 2). In addition, the seeds shell usually covers not all surface of a kernel, and the kernel usually gets all space restricting by the shell. Overall, the ‘rice’ type is characterized by minimal seed shell rate.

Tartary buckwheat accessions with ‘round’ type of seeds (recessive homozygous at *SMK*) showed wide variation in the seed shell rate together with seed shell thickness (Table 2). ‘Notched’ type (wild type for both *SMK* and *SPL*) unexpectedly manifested similar range of variation for both the parameters.

## Discussion

Although Tartary buckwheat grain is better than common buckwheat grain as a raw material for the production of whole-grain flour (Klimova *et al.* 2020), the groats production, i.e. shelling of Tartary buckwheat grain, has some peculiarities and can be problematic. The latter strongly depends on characteristics of the grain, and, to a certain extent, on their size (Li *et al.* 2019, Wang and Campbell 2007).

Among polymorphism of *F. tataricum* by type of seeds, for groats production the rice-type is best suited (Li *et al.* 2019, Mukasa *et al.* 2009, Wang and Campbell 2007). However, such forms are very small-grained, even in comparison with other samples of this rather small-grained species with maximal value of thousand seed weight (TSW) about 23 g (at diploid level). Available samples of the rice type show the TSW in the range 11.7–17.7 g, sometimes less than 10 g (Li *et al.* 2019). To compare, cultivated *F. esculentum* varies in the weight of 1000 seeds in a very wide range (23–39 g); the optimum falls in the range of 28–30 g (Fesenko *et al.* 2015). The small size of Tartary buckwheat groats compared to one of common buckwheat in some way may be useful for marketing as it provides a good distinctiveness of the product with better properties. However, some organizational or technical problems associated with the processing of such grain on the equipment used for the production of groats from common buckwheat grain are not excluded.

An effective approach to significantly increase TSW is interspecific hybridization. In this way, the species *F. hybridum* was obtained in cross *F. tataricum* × *F. giganteum*. The species manifests TSW = 41.1 g. In addition, in crosses *F. cymosum* × *F. giganteum* the plants with TSW of about 30 g were obtained. Apparently, this approach is also promising for the regulation of TSW in varieties based on Tartary buckwheat.

Another aspect of the use of interspecific hybrids of *F. tataricum* with *F. cymosum* is the transfer of genes that control the triangular (cymosum-like) seed shape into *F. tataricum* genome, i.e. the synthesis of plant material combining the seed characteristics of *F. cymosum* with growing season characteristics of *F. tataricum*. This would make it possible to quickly introduce such material into the industry specializing in the groats production. At present, we have not yet obtained forms that combine the large triangular grain with the optimal growing season characteristics. However, data were obtained on a simple genetic control of this type of seeds (Fesenko 2012). In this work we used the forms with a share of *F. tataricum* genome near 12%. Future work will show possibility to increase the share and to make this material earlier-ripening. Finally, our work revealed a functional allelism of the mutations resulting in triangular type of seeds in both *F. cymosum* and *F. esculentum*. Thus, when the gene that determines the seeds type of *F. cymosum* will be transferred into the genome of *F. tataricum*, it will be obtained the Tartary buckwheat with seeds as in common buckwheat, and on the same genetic basis.

One more significant parameter in some cases is seed shell rate which varies within *F. tataricum*. Mutations and their combinations which determine the type of seeds affect this trait. The thinnest shell is a characteristic of the rice type. However, within the same type of seed this variation also can be observed. So, among F_2_ fractions with rice and round types of seeds the ranges of the variation were 2 times (for rice type) and 3 times (for round type) wider than among parental accessions of same types (Li *et al.* 2019). In addition, among F_2_ hybrids of round-type there were plants with both more thick and more thin shell compared to the round-type parent; among F_2_ hybrids of rice type there were not plants with thinner shell compared to the rice-type parent (Li *et al.* 2019). It suggests the additional genes regulating the seed shell thickness regardless genotype for the genes determining seed type. It was supported by analyzing of some interspecific hybrids of buckwheat in our work. We obtained a wide variation within the ten of BC_1_F_2_ *F. cymosum* × *F. giganteum* hybrids forming seeds of same type with *F. cymosum*. Among the hybrids were both plants that form seeds with a thin shell, which almost did not differ from that of *F. cymosum*, and plants that form seeds with a thicker shell. Also, *F. hybridum* forms seeds with a more massive shell than the k-108 *F. tataricum* line despite the same genotype for genes determining the type of seeds. Finally, the hybrid *F. esculentum* × *F. cymosum* formed the seed shell that was thicker than the both parental species. It all seems to indicate that different species, as well as different accessions within same species, carry different combinations of multiple genes involved in controlling the thickness of the seed coat. Comprehensive genetic analysis of this trait was not yet conducted. But the experimental results, mainly those on segregation among the BC_1_F_2_ *F. cymosum* × *F. giganteum* hybrids in our work, and also on segregation among F_2_ hybrids between accessions of *F. tataricum* published by Li *et al.* (2019), show the possibility of selection for this trait, if it is necessary.

## Author Contribution Statement

IF designed the experiment, conducted interspecific crosses and wrote the paper. NB conducted embryo rescue and *in vitro* cultivation of interspecific hybrids. OR and DE evaluated the seeds characteristics. IF and AF analyzed and interpreted the data. All authors have read and agreed to the published version of the manuscript.

## Figures and Tables

**Table 1.** Thousand seeds weight and seed shell rate of *Fagopyrum tataricum* (FT), *F. cymosum* FC, *F. esculentum* (FE), *F. giganteum* (FG), *F. hybridum* (FH), and interspecific hybrids

Seeds’ type	Species, hybrid	n	Thousand seeds weight, g (range)	Seed shell rate, % (range)
Notched	FT, 2x	15	16.1–22.5	21.9–30.2
	FG	1	27	32.5
Round	FT, 2x	4	16.2–20.7	21.6–30.1
	FT, 4x	1	25.5	35.29
	FH	1	41.1	30.7
Rice	FT, 2x	10	11.7–17.7	13.6–18.4
Triangular	FE, 2x	4	28.7–33.2	19.6–24.7
	FE, 4x	1	42.6	26.4
	FC, 4x	1	20.3	21.5
	BC_1_F_2_ FC × FG	9*^a^*	16.0–29.9	26.7–32.9

*^a^* Number of hybrid plants analyzed.

**Table 2.** Seed shell thickness (μm) of *Fagopyrum tataricum* (FT), *F. cymosum* FC, *F. esculentum* (FE), *F. giganteum* (FG), *F. hybridum* (FH), and interspecific hybrids

Seeds’ type	Species, hybrid	Number of		X ± SE	Range
		accessions/hybrid plants	seeds		
Notched	FT, 2x	15	30	105.7 ± 4.4	80.1–124.8
	FG	1	10	141.9 ± 6.1	102.7–187.7
Round	FT, 2x	4	20	103.9 ± 3.0	73.6–130.8
	FT, 4x	1	10	111.4 ± 5.5	98.0–120.3
	FH	1	10	135.1 ± 3.9	100.7–167.7
Rice	FT, 2x	10	20	55.1 ± 0.8	49.0–57.7
Triangular	FE, 2x	4	20	70.0 ± 1.2	46.7–83.3
	FE, 4x	1	10	69.0 ± 2.1	44.3–87.7
	FC, 4x	1	10	88.8 ± 2.9	83.7–95.3
	F_1_ FE × FC, 4x	2*^a^*	2	99.6	97.9–101.3
	BC_1_F_2_ FC × FG	9*^b^*	45	107.8 ± 3.5	86.6–151.2

*^a^* Number of seeds analyzed. *^b^* Number of hybrid plants analyzed.
